# Breaking immune tolerance by targeting Foxp3^+^ regulatory T cells mitigates Alzheimer's disease pathology

**DOI:** 10.1038/ncomms8967

**Published:** 2015-08-18

**Authors:** Kuti Baruch, Neta Rosenzweig, Alexander Kertser, Aleksandra Deczkowska, Alaa Mohammad Sharif, Amit Spinrad, Afroditi Tsitsou-Kampeli, Ayelet Sarel, Liora Cahalon, Michal Schwartz

**Affiliations:** 1 Department of Neurobiology, Weizmann Institute of Science, 234 Herzl Street, Rehovot 76100, Israel

## Abstract

Alzheimer's disease (AD) is a neurodegenerative disorder in which chronic neuroinflammation contributes to disease escalation. Nevertheless, while immunosuppressive drugs have repeatedly failed in treating this disease, recruitment of myeloid cells to the CNS was shown to play a reparative role in animal models. Here we show, using the 5XFAD AD mouse model, that transient depletion of Foxp3^+^ regulatory T cells (Tregs), or pharmacological inhibition of their activity, is followed by amyloid-β plaque clearance, mitigation of the neuroinflammatory response and reversal of cognitive decline. We further show that transient Treg depletion affects the brain's choroid plexus, a selective gateway for immune cell trafficking to the CNS, and is associated with subsequent recruitment of immunoregulatory cells, including monocyte-derived macrophages and Tregs, to cerebral sites of plaque pathology. Our findings suggest targeting Treg-mediated systemic immunosuppression for treating AD.

## Abstract (web summary)

Immunosuppression has been unsuccessful in treatment of Alzheimer's disease. Here the authors show in a mouse model of the disease that transient inhibition of regulatory T cells mitigates amyloid plaque pathology and reverses cognitive decline, whereas augmenting these cells worsens the pathology.

## Graphical abstract

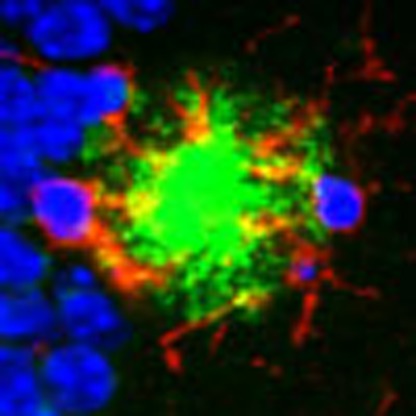

Alzheimer's disease (AD) is an age-related neurodegenerative condition characterized by neuronal damage, amyloid-beta (Aβ) plaque deposition and chronic inflammation within the central nervous system (CNS), leading to gradual loss of cognitive function and brain tissue destruction^1^,^2^,^3^. Inconsistent findings of clinical and pre-clinical studies over the past decade, investigating AD therapies based on immunosuppression, have raised the question of why anti-inflammatory drugs fall short in AD^4^,^5^,^6^,^7^.

Multiple lines of evidence suggest that under neurodegenerative conditions in general, and in AD in particular, circulating myeloid cells, and the resident myeloid cells of the CNS, the microglia, play non-redundant roles in mitigating the neuroinflammatory response^8^,^9^,^10^. Specifically, while microglia fail to ultimately clear Aβ deposits, CNS-infiltrating monocyte-derived macrophages (mo-MΦ) play a beneficial role in facilitating Aβ plaque removal and fighting off AD-like pathology^11^,^12^,^13^,^14^,^15^,^16^.

Our group has recently pointed to the brain's choroid plexus (CP), the epithelial layer that forms the blood–CSF barrier, as a selective gateway for leukocyte entry to the CNS, enabling recruitment of mo-MΦ and T cells following neural tissue injury^17^,^18^, and in neurodegeneration^19^,^20^. In addition, we suggested that in analogy to the situation in cancer immunology, onset of clinical symptoms in neurodegenerative diseases might reflect the loss of immune surveillance^21^, and that systemic immune suppression might impair the ability to mount an immune response needed for fighting brain pathology^22^,^23^. Accordingly, in the present study we hypothesized that in AD suboptimal recruitment of inflammation-resolving immune cells to the diseased brain parenchyma may be an outcome of systemic immune failure, involving CP gateway dysfunction. Here we identify Treg-mediated systemic immune suppression as a negative player in AD pathology, and demonstrate how targeting Tregs in AD-Tg mice augments interferon (IFN)-γ-dependent CP gateway activity, leading to accumulation of inflammation-resolving immune cells at cerebral sites of pathology and disease mitigation.

## Results

### CP gateway dysfunction in AD-Tg mice

**Figure 1:**
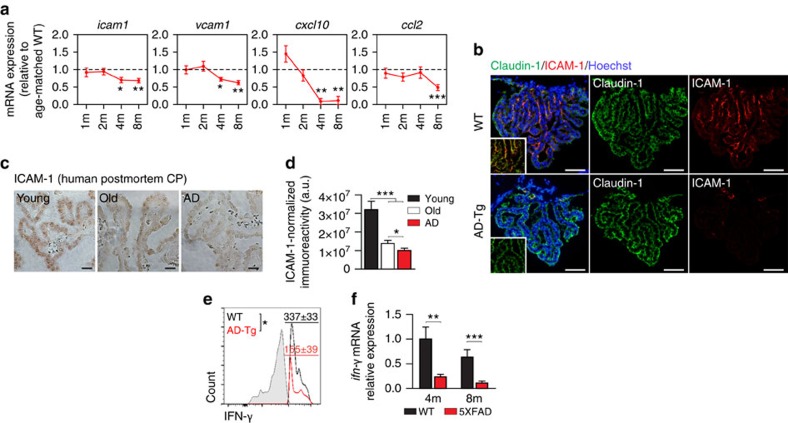
Choroid plexus gateway dysfunction in AD-Tg mice. (**a**) mRNA expression levels for the genes *icam1*, *vcam1*, *cxcl10* and *ccl2*, measured by RT-qPCR, in CPs isolated from 1-, 2-, 4- and 8-month-old AD-Tg mice, shown as fold-change compared with age-matched WT controls (*n*=6–8 per group; Student's *t*-test for each time point). (**b**) Representative microscopic images of CPs of 8-month-old AD-Tg mice and age-matched WT controls, immunostained for the epithelial tight junction molecule Claudin-1 (green), Hoechst nuclear staining (blue) and the integrin ligand, ICAM-1 (red; scale bar, 50 μm). Inserts showing Claudin-1 (green) and ICAM-1 (red) double staining. (**c**–**d**) Representative micrographs (**c**), and quantification (**d**), of ICAM-1 immunoreactivity in human postmortem CP of young and aged non-CNS-diseased and AD patients (scale bar, 50 μm). (**e**) Flow cytometry analysis of IFN-γ-expressing immune cells (intracellularly stained, and pre-gated on CD45) in CPs of 8-month-old AD-Tg mice and age-matched WT controls. Shaded histogram represents isotype control (*n*=4–6 per group; Student's *t*-test). (**f**) mRNA expression levels of *ifn-γ*, measured by RT-qPCR, in CP tissues isolated from 4- and 8-month-old AD-Tg mice, compared with age-matched WT controls (*n*=5–8 per group; Student's *t*-test for each time point). In all panels, error bars represent mean±s.e.m.; *P<0.05; **P<0.01; ***P<0.001.

We first examined CP activity in supporting leukocyte trafficking to the CNS along disease progression in the 5XFAD transgenic mouse model of AD (AD-Tg); these mice co-express five mutations associated with familial AD, and develop cerebral Aβ pathology and gliosis as early as 2 months of age^24^. We found that along the progressive stages of disease pathology, the CP of AD-Tg mice, compared with age-matched wild-type (WT) controls, expressed significantly lower levels of leukocyte homing and trafficking determinants, including intercellular adhesion molecule 1 (*icam1)*, vascular cell adhesion molecule 1 (*vcam1*), C-X-C motif chemokine 10 (*cxcl10*) and chemokine C-C motif ligand 2 (*ccl2*) (Fig. 1a), shown to be upregulated by the CP in response to acute CNS damage and needed for transepithelial migration of leukocytes^17^,^18^,^19^. Immunohistochemical staining for the integrin ligand, ICAM-1, confirmed its reduced expression by the CP epithelium of AD-Tg mice (Fig. 1b). In addition, staining for ICAM-1 in human postmortem brains, showed its age-associated reduction in the CP epithelium, in line with our previous observations^25^, and quantitative assessment of this effect revealed further decline in AD patients compared with aged individuals without CNS disease (Fig. 1c,d). Since the induction of leukocyte trafficking determinants by the CP is dependent on epithelial IFN-γ signalling^17^, and IFN-γ levels at the CP were found to be reduced in brain aging^25^,^26^ and under neurodegenerative conditions^20^, we next-tested whether the observed effects in AD-Tg mice could reflect loss of IFN-γ availability at the CP. Examining the CP of 5XFAD AD-Tg mice using intracellular staining followed by flow cytometry, revealed significantly lower numbers of IFN-γ-producing cells in this compartment (Fig. 1e), and quantitative real-time PCR (RT-qPCR) analysis confirmed lower mRNA expression levels of *ifn-γ* at the CP of AD-Tg mice compared with age-matched WT controls (Fig. 1f).

### Transient depletion of Foxp3^+^ Tregs mitigates AD pathology

Regulatory T cells (Tregs) play a pivotal role in suppressing systemic effector immune responses and maintenance of immune tolerance^27^. We therefore envisioned that reduced IFN-γ availability at the CP of AD-Tg mice might be affected by systemic immune suppression, and focused on the involvement of Tregs in AD pathology. Notably, we found elevated levels of Foxp3^+^ Treg along disease progression in splenocytes of 5XFAD AD-Tg mice, relative to age-matched WT littermates (Supplementary Fig. 1a,b), in line with reports of elevated Treg levels and their suppressive activities in AD patients^28^,^29^,^30^.

**Figure 2:**
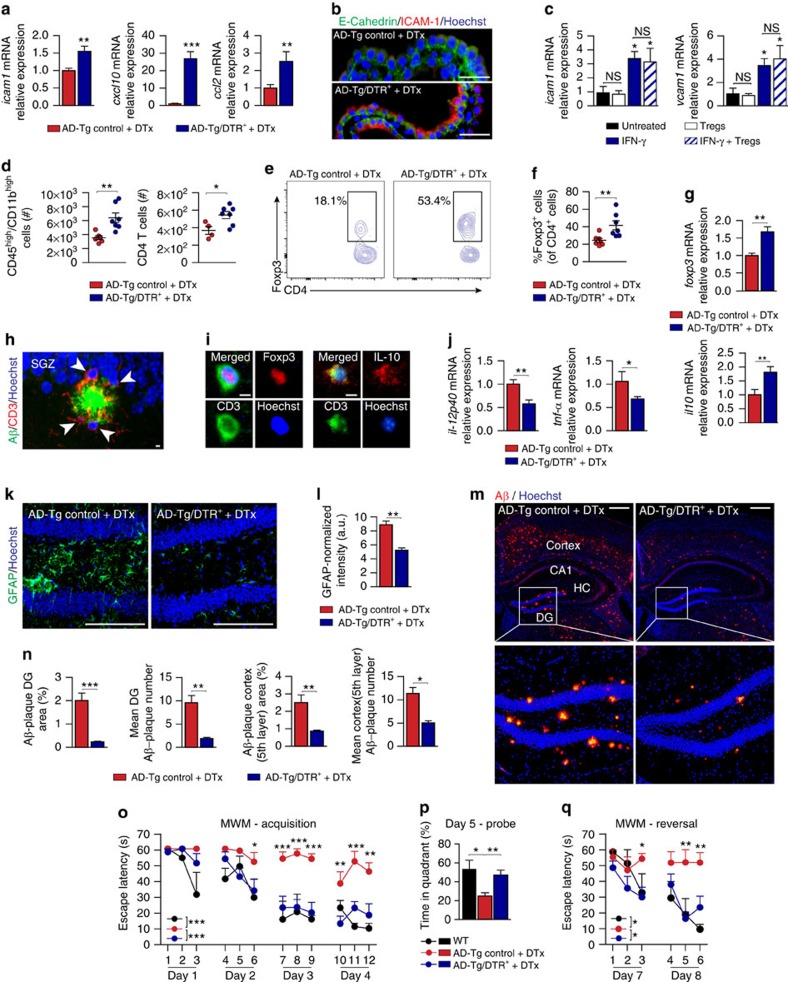
Transient conditional depletion of Foxp3^+^ Tregs activates the CP for leukocyte trafficking and mitigates AD pathology. (**a**) AD-Tg/Foxp3-DTR^+^ and non-DTR-expressing AD-Tg littermates (AD-Tg/Foxp3-DTR^−^; AD-Tg control) were treated with DTx for 4 consecutive days. mRNA levels of *icam1*, *cxcl10* and *ccl2* in the CP of 6-month-old AD-Tg/Foxp3-DTR^+^ mice, 1 day after last injection (*n*=6–8 per group; Student's *t*-test). (**b**) Representative images of the CP, immunostained for E-Cadherin (green), ICAM-1 (red) and Hoechst, in 6-month-old AD-Tg/Foxp3-DTR^+^ mice, 1 day after last DTx injection (Scale bar, 25 μm). (**c**) mRNA levels of *icam1* and *vcam1*, in cultured CP cells, 24 h after addition of *ex vivo* differentiated Tregs, IFN-γ or their combination, relative to untreated (UT) cells (*n*=3–4 per group; one-way ANOVA followed by Newman–Keuls *post hoc* test; NS, not significant). (**d**–**f**) Flow cytometry analysis of the brain of 6-month-old AD-Tg/Foxp3-DTR^+^ mice, 3 weeks following last DTx injection, showing increased numbers of CD11b^high^/CD45^high^ mo-MΦ and CD4^+^ T cells (**d**), and increased CD4^+^Foxp3^+^ (**e**,**f**) Treg frequencies (*n*=4–7 per group; Student's *t*-test). (**g**) mRNA levels of *foxp3* and *il10* in the brain of 6-month-old AD-Tg/Foxp3-DTR^+^ mice, 3 weeks after last DTx injection (*n*=6–8 per group; Student's *t*-test). (**h**,**i**) Representative images of Aβ (green) and CD3 (red) (**h**), or Foxp3 and IL-10 (in red) immunostaining (**i**) in 6-month-old AD-Tg/Foxp3-DTR^+^, 3 weeks following last DTx injection (scale bar, 10 μm). (**j**) mRNA levels of *il-12p40* and *tnf-a* in the brain of 6-month-old AD-Tg/Foxp3-DTR^+^ mice, 3 weeks after last DTx injection (*n*=6–8 per group; Student's *t*-test). (**k**,**l**) Representative images (**k**) and analysis (**l**) of GFAP immunostaining in hippocampal sections of 6-month-old AD-Tg/Foxp3-DTR^+^ mice, 3 weeks following the last DTx injection (scale bar, 250 μm; *n*=3–5 per group; Student's *t*-test). (**m**,**n**) Representative images (**m**) and analysis (**n**), of 5-month-old AD-Tg/Foxp3-DTR^+^ mice brains, immunostained for Aβ (in red) and Hoechst, 3 weeks after the last DTx injection (scale bar, 250 μm). Mean Aβ area and plaque numbers in the dentate gyrus (DG) and the cortex fifth layer (*n*=5–6 per group; Student's *t*-test). (**o**–**q**) Morris water maze (MWM) of 6-month-old AD-Tg/Foxp3-DTR^+^, 3 weeks after last DTx injection. DTx-treated mice showed improved spatial learning/memory in the acquisition (**o**), probe (**p**) and reversal (**q**) phases, relative to AD-Tg controls (*n*=7–9 per group; two-way repeated measures ANOVA followed by Bonferroni *post hoc* test; *P<0.05 for overall acquisition, probe and reversal). In all panels, error bars represent mean±s.e.m.; *P<0.05; **P<0.01; ***P<0.001.

To study the functional relationships between Treg-mediated systemic immune suppression, CP gateway activity and AD pathology, we cross-bred 5XFAD AD-Tg mice with Foxp3-diphtheria toxin (DTx) receptor (DTR^+^) mice, enabling transient conditional *in vivo* depletion of Foxp3^+^ Tregs^31^ in AD-Tg/DTR^+^ mice by administration of DTx (Supplementary Fig. 1c). We found that transient depletion of Tregs resulted in elevated mRNA expression levels of leukocyte trafficking molecules by the CP of AD-Tg/DTR^+^ mice, relative to DTx-treated AD-Tg/DTR^−^ littermates (Fig. 2a), and immunohistochemistry confirmed elevated ICAM-1 immunoreactivity (Fig. 2b). These findings suggested that the mechanism whereby Treg depletion affected CP activity involves increased IFN-γ availability in this compartment. We also tested whether alternatively, interleukin-10 (IL-10), a Treg-secreted cytokine, or Treg cells could potentially act on CP epithelial cells, either directly or by suppressing the effect of IFN-γ. We found no evidence for a direct effect of IL-10 (Supplementary Fig. 1d) or Tregs (Fig. 2c) on the response of cultured CP cells to IFN-γ. Previous findings in other epithelial barrier tissues also showed lack of response to IL-10 (ref. 32). These findings suggest that the Treg-mediated effect on CP function is not direct.

To examine how activation of this immune-brain axis affected disease pathology, we examined AD-Tg mice 3 weeks following the Treg depletion, to allow detection of the consequent effect of CP activation for leukocyte trafficking to the CNS. We observed immune cell accumulation in the brain, including elevated numbers of CD45^high^/CD11b^high^ myeloid cells, representing infiltrating mo-MΦ^18^ and CD4^+^ T cells (Fig. 2d; Supplementary Fig. 1e). In addition, brief and transient depletion of Tregs resulted in a marked enrichment of Foxp3^+^ Tregs among the CD4^+^ T cells that accumulated within the brain, as assessed by flow cytometry (Fig. 2e,f). RT-qPCR analysis of the hippocampus showed increased expression of *foxp3* and *il10* mRNA levels (Fig. 2g), and immunohistochemical analysis revealed T cells adjacent to Aβ plaques (Fig. 2h); among these were IL-10- and Foxp3-expressing cells (Fig. 2i). Thus, transiently breaking Treg-mediated systemic immune suppression in AD-Tg mice, augmented the recruitment of inflammation-resolving immune cells, including mo-MΦ and Tregs, to cerebral sites of Aβ pathology.

We next evaluated whether the transient depletion of Tregs in AD-Tg mice, which resulted in accumulation of immunoregulatory cells in the brain parenchyma, led to an effect on the local neuroinflammatory response, plaque pathology and cognitive function. Examining the hippocampal cytokine milieu by RT-qPCR, revealed reduced mRNA expression levels of the pro-inflammatory cytokines, *il-12p40* and *tnf-α* (Fig. 2j), and immunohistochemical analysis showed a reduction in gliosis (Fig. 2k,l). Moreover, cerebral Aβ plaque burden in the hippocampal dentate gyrus (DG) and the cerebral cortex (fifth layer), two brain regions exhibiting robust Aβ plaque pathology in 5XFAD AD-Tg mice^24^, was reduced (Fig. 2m,n). Next, we evaluated the effect on spatial learning and memory performance using the Morris water maze test, and found a significant improvement in cognitive function in AD-Tg/DTR^+^ mice following Treg depletion, relative to DTx-treated AD-Tg/DTR^−^ age-matched controls, reaching performance similar to that of WT mice (Fig. 2o–q). Taken together, these results demonstrated that transiently breaking Treg-mediated systemic immune suppression in AD-Tg mice initiated a time-dependent cascade of events, which involved augmented recruitment of immune cells to the brain parenchyma, and culminated in attenuation of the neuroinflammatory response, clearance of Aβ plaques and reversal of cognitive decline.

### Activation of the CP in AD-Tg mice by immunomodulation

**Figure 3:**
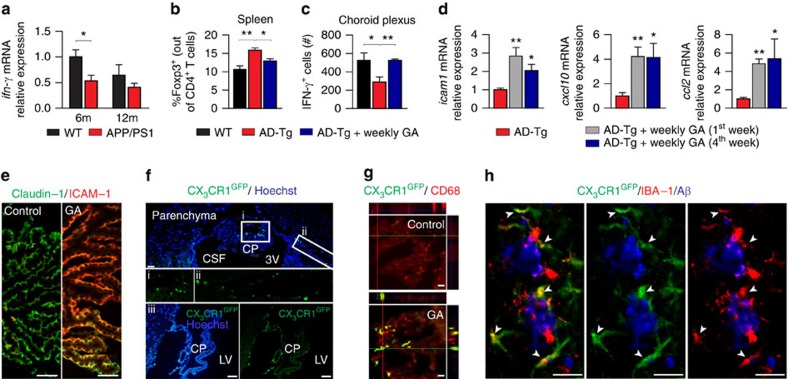
Immunomodulation that reduces systemic Treg levels activates the CP for mo-MΦ trafficking and mitigates AD pathology. (**a**) mRNA expression levels of *ifn-γ*, measured by RT-qPCR, in CPs isolated from 6- and 12-month-old APP/PS1 AD-Tg mice, compared with age-matched WT controls (*n*=5–8 per group; Student's *t*-test). (**b**,**c**) 5XFAD AD-Tg mice were treated with either weekly GA or vehicle (PBS), and were examined at the end of the 1st week of the administration regimen (after a total of two GA injections). Flow cytometry analysis of CD4^+^Foxp3^+^ splenocyte frequencies (**b**) and CP-resident IFN-γ-expressing immune cells (intracellularly stained and pre-gated on CD45) (**c**), in treated 6-month-old AD-Tg mice, compared with age-matched WT controls (*n*=4–6 per group; one-way ANOVA followed by Newman–Keuls *post hoc* analysis). (**d**) mRNA expression levels for the genes *icam1*, *cxcl10* and *ccl2*, measured by RT-qPCR, in CPs of 4-month-old AD-Tg mice, treated with either weekly GA or vehicle, and examined either at the end of the 1st or 4th week of the weekly GA regimen (*n*=6–8 per group; one-way ANOVA followed by Newman–Keuls *post hoc* analysis). (**e**) Representative microscopic images of 6-month-old AD-Tg mice following weekly GA, stained for ICAM-1 (in red) and Claudin-1 (in green; epithelial tight junctions), showing elevated levels of ICAM-1 immunoreactivity, as compared with vehicle-treated AD-Tg (scale bar, 50 μm). (**f**) Representative images of brain sections from 6-month-old AD-Tg/CX_3_CR1^GFP/+^ BM chimeras following weekly GA. CX_3_CR1^GFP^ cells were localized at the CP of the third ventricle (3V; i), the adjacent ventricular spaces (ii), and the CP of the lateral ventricles (LV; iii) in AD-Tg mice treated with weekly GA (scale bar, 25 μm). (**g**) Representative orthogonal projections of confocal *z*-axis stacks, showing co-localization of GFP^+^ cells (in green) with the myeloid marker, CD68 (in red), in the CP of 7-month-old AD-Tg/CX_3_CR1^GFP/+^ mice treated with weekly GA, but not in control PBS-treated AD-Tg/CX_3_CR1^GFP/+^ mice (scale bar, 25 μm). (**h**) CX_3_CR1^GFP^ cells (in green) are co-localized with the myeloid marker IBA-1 in brains of GA-treated AD-Tg/CX_3_CR1^GFP/+^ mice in the vicinity of Aβ plaques, and co-express the myeloid marker, IBA-1 (in red). Arrowheads indicate co-labelled IBA-1^+^/GFP^+^ cells (scale bar, 25 μm). In all panels, error bars represent mean±s.e.m.; *P<0.05; **P<0.01; ***P<0.001.

We next-tested whether the immunomodulatory compound, glatiramer acetate (GA; also known as Copolymer-1 or Copaxone), which was found in a weekly administration regimen to have a therapeutic effect in the APP/PS1 mouse model of AD^33^, associated with mo-MΦ recruitment to cerebral sites of disease pathology^12^,^16^, would induce CP activation to enhance leukocyte trafficking. We found that the CP in APP/PS1 AD-Tg mice expresses lower levels of IFN-γ relative to age-matched WT controls (Fig. 3a), similarly to our observation in the 5XFAD AD-Tg mouse model (Fig. 1f). These findings encouraged us to test whether the observed therapeutic effect of weekly administration of GA in APP/PS1 mice^33^ could be reproduced in 5XFAD AD-Tg mice, and if so, whether it would involve an effect on systemic Tregs and IFN-γ-activation of the CP for mo-MΦ trafficking. We therefore treated 5XFAD AD-Tg mice with a weekly administration regimen of GA over a period of 4 weeks (henceforth, ‘weekly GA'; schematically depicted in Supplementary Fig. 2a). We found that 5XFAD AD-Tg mice treated with weekly GA showed reduced neuroinflammation (Supplementary Fig. 2b,c), enhanced hippocampal expression of brain-derived neurotrophic factor (*bdnf*) and insulin-like growth factor-1 (*igf1*) (Supplementary Fig. 2d) and improved cognitive performance (Supplementary Fig. 2e–g), which lasted up to 2 months after the treatment (Supplementary Fig. 2h,i). Examining by flow cytometry the effect of weekly GA on systemic immunity and on the CP, we found reduced splenocyte Foxp3^+^ Treg levels (Fig. 3b), and an increase in IFN-γ-producing cells at the CP of the treated 5XFAD AD-Tg mice, reaching similar levels to those observed in WT controls (Fig. 3c). The elevated numbers of IFN-γ-expressing cells in the CP of weekly GA-treated mice correlated with the upregulated expression of leukocyte trafficking molecules by the CP (Fig. 3d). Immunohistochemical analysis of the CP, in the weekly GA-treated AD-Tg mice, confirmed increased levels of epithelial ICAM-1 (Fig. 3e), and showed altered epithelial tight junction organization (Supplementary Fig. 3a), previously associated with CNS-infiltrating mo-MΦ trafficking through the CP-CSF migratory pathway^17^,^18^,^20^.

To detect infiltrating mo-MΦ entry to the CNS, we used 5XFAD AD-Tg/CX_3_CR1^GFP/+^ bone marrow (BM) chimeric mice (prepared using head protection; see Methods), allowing the visualization of circulating (green fluorescent protein (GFP)^+^ labelled) myeloid cells^18^,^20^. We found increased homing of GFP^+^ mo-MΦ to the CP and to the adjacent ventricular spaces following weekly GA treatment, as compared with vehicle-treated AD-Tg/CX_3_CR1^GFP/+^ controls (Fig. 3f,g; Supplementary Fig. 3b), and immunohistochemistry of the brain parenchyma revealed the accumulation of GFP^+^ mo-MΦ at cerebral sites of plaque formation (Fig. 3h). These findings were further confirmed in non-chimeric AD-Tg mice, by flow cytometry analysis of the hippocampus, which showed increased numbers of CD11b^high^CD45^high^-expressing cells (Supplementary Fig. 3c,d), representing a myeloid population enriched with CNS-infiltrating mo-MΦ^18^. Examination of the blood–brain barrier (BBB) endothelial basal lamina, in weekly GA-treated and -untreated 5XFAD AD-Tg mice (Supplementary Fig. 3e) showed no signs of perivascular cuffing associated with BBB breakdown and endothelial leukocyte transmigration^34^. These results are consistent with our present working hypothesis of the functional linkage between previously observed mo-MΦ recruitment to sites of AD pathology and IFN-γ-dependent activation of the CP.

**Figure 4:**
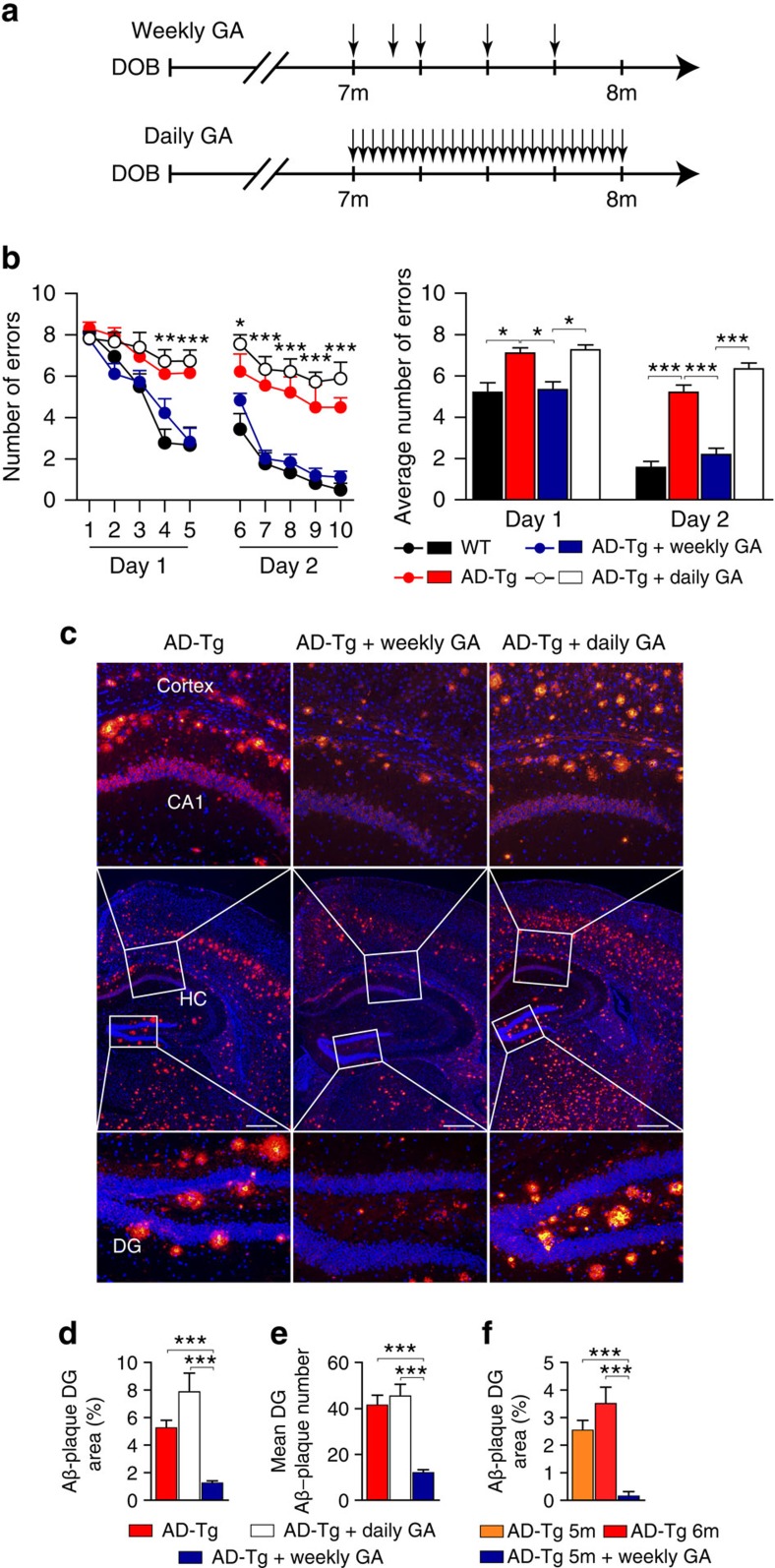
Distinct effects of weekly versus daily-administration regimens of GA on disease pathology and cognitive performance in AD-Tg mice. (**a**) Schematic representation of daily-GA treatment regimen compared with the weekly GA regimen. In the daily-GA-treated group, mice were s.c. injected daily with 100 μg of GA for a period of 1 month (DOB, day of birth). (**b**) Cognitive performance of daily-GA and weekly GA-treated 7-month-old AD-Tg mice, compared with age-matched WT and untreated AD-Tg mice, as assessed by the average numbers of errors per day in the RAWM learning and memory task (*n*=6–8 per group; two-way repeated measures ANOVA followed by Bonferroni *post hoc* analysis, and one-way ANOVA followed by Newman–Keuls *post hoc* analysis for individual pair comparisons of average numbers of errors). (**c**) Representative microscopic images of the cerebral cortex and the hippocampus (HC) of untreated AD-Tg, and daily or weekly GA-treated AD-Tg mice, immunostained for Aβ plaques (red) and with Hoechst nuclear staining (blue) (scale bar, 250 μm). (**d**,**e**) Quantification of Aβ plaque size and numbers (per 6-μm slices) in GA-treated (daily-GA and weekly GA groups) and untreated AD-Tg mice. Weekly GA-treated AD-Tg mice showed reduction in Aβ plaque load as a percentage of the total area of their hippocampal dentate gyrus (DG), and in mean Aβ plaque numbers (*n*=6 per group; one-way ANOVA followed by Newman–Keuls *post hoc* analysis). (**f**) Five-month-old AD-Tg were either left untreated for 1 month, and evaluated for their plaque pathology, or treated for 1 month with weekly GA and then examined. Mean Aβ plaque area as percentage of the dentate gyrus showed clearance of Aβ plaques in the GA-treated mice group (*n*=3–4 per group; one-way ANOVA followed by Newman–Keuls *post hoc* analysis). In all panels, error bars represent mean±s.e.m.; *P<0.05; **P<0.01; ***P<0.001.

Daily injections of GA, both in the clinic for treating relapsing-remitting multiple sclerosis and in animal studies induce Treg-based peripheral immune suppression^35^,^36^,^37^. Given our present findings of the negative effect of systemic Tregs on AD pathology, and reduced systemic Treg levels following a weekly administration regimen of GA, we envisioned that comparing the effect of GA in these two administration regimens (weekly versus daily) might further contribute to the understanding of the inverse relationship between systemic Tregs and AD progression. We therefore compared the effect of daily- versus weekly-GA administration over a period of 4 weeks (schematically depicted in Fig. 4a), on disease pathology in 5XFAD AD-Tg mice. Assessment of cognitive performance by the radial-arm water maze (RAWM) task revealed that, in contrast to the beneficial effect of weekly GA treatment over a period of 4 weeks, either no beneficial effect on spatial memory or a tendency towards a worsening effect was observed in AD-Tg mice that received daily-GA (Fig. 4b). In addition, unlike the robust effect of the weekly GA administration on plaque clearance, daily-GA-treated AD-Tg mice did not show any beneficial effect, or showed a moderate adverse effect on plaque load (Fig. 4c–e). Importantly, our results further showed that weekly GA administration not only arrested plaque formation, but reversed disease pathophysiology in terms of Aβ plaque burden (Fig. 4f).

### Disease attenuation by interfering with Foxp3^+^ Treg activity

**Figure 5:**
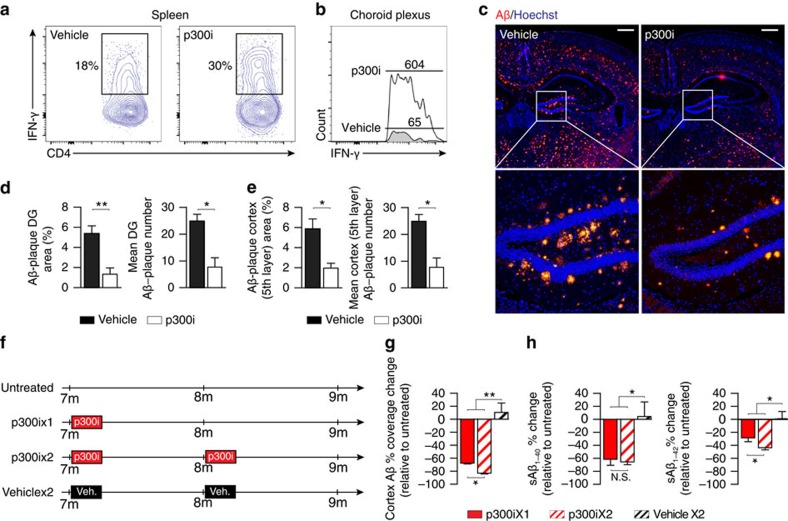
Interference with Foxp3^+^ Treg cell activity using p300 inhibitor mitigates AD pathology. (**a**,**b**) Aged WT mice (18 months) were used to test the effect of p300i on IFN-γ-expressing cell levels in the CP and spleen. Mice were treated with either p300i or vehicle (DMSO) for a period of 1 week, and examined a day after cessation of treatment. Representative flow cytometry plots showing elevation in the frequencies of CD4^+^ T cells expressing IFN-γ in the spleen (**a**), and IFN-γ-expressing immune cell numbers in the CP (**b**), following p300i treatment. (**c**–**e**) Representative microscopic images (**c**), and quantitative analysis, of Aβ plaque burden in the brains of 10-month-old AD-Tg mice that received either p300i or vehicle (DMSO) for a period of 1 week, and were subsequently examined after 3 additional weeks. Brains were immunostained for Aβ plaques (red) and by Hoechst nuclear staining (*n*=5 per group; Scale bar, 250 μm). Mean Aβ plaque area and plaque numbers were quantified in the hippocampal DG (**d**) and the fifth layer of the cerebral cortex (**e**), in 6 μm brain slices (*n*=5–6 per group; Student's *t*-test). (**f**–**h**) Schematic representation (**f**) of the p300i treatment (or DMSO, vehicle) regimen to the different groups of AD-Tg mice at the age of 7 months, in either one or two treatment courses. Change in mean of Aβ plaque percentage coverage of the cerebral cortex (fifth layer) (**g**), and the change in mean cerebral soluble Aβ_1-40_ and Aβ_1-42_ protein levels (**h**), relative to the untreated AD-Tg group (Aβ_1-40_ and Aβ_1-42_ mean level in untreated group, 90.5±11.2 and 63.8±6.8 pg/mg total portion, respectively; *n*=5–6 per group; one-way ANOVA followed by Newman–Keuls *post hoc* analysis). In all panels, error bars represent mean±s.e.m.; *P<0.05; **P<0.01.

The findings above, which suggested that Treg-mediated systemic immune suppression interferes with the ability to fight AD pathology, are reminiscent of the function attributed to Tregs in cancer immunology, in which these cells hinder the ability of the immune system to mount an effective anti-tumour response^38^,^39^,^40^. We therefore tested p300i (C646), a nonpeptidic inhibitor of the histone acetyltransferase p300 (ref. 41); although histone acetyltransferases can have a direct effect on the brain^42^, p300i was found to regulate T-cell function by impairing Treg suppressive activities without affecting effector T-cell protective responses^43^. We found that mice treated with p300i, compared with vehicle (dimethylsulphoxide (DMSO))-treated controls, showed elevated levels of systemic IFN-γ-expressing cells in the spleen (Fig. 5a), as well as in the CP (Fig. 5b). We next treated AD-Tg mice at the age of 10 months (a stage of robust cerebral plaque pathology) with p300i over a course of 1 week, and examined the mice 3 weeks later for cerebral Aβ plaque burden. Immunohistochemical analysis revealed a significant reduction in cerebral Aβ plaque load in the p300i-treated AD-Tg mice relative to vehicle-treated controls (Fig. 5c–e). We also tested whether the effect on plaque pathology following a single course of treatment would last beyond 3 weeks, and if so, whether additional courses of treatment would be effective over a longer period of time. We compared AD-Tg mice that received a single course of p300i treatment and were examined 2 months later, to an age-matched group that received two treatment courses during this period, with a 1-month interval in between (schematically depicted in Fig. 5f). We found that the reduction of cerebral plaque load was evident even 2 months after a single course of treatment, but was stronger in mice that received two courses of treatment at a 1-month interval (Fig. 5g).

Since impaired synaptic plasticity and memory deficits in AD are associated with elevated cerebral soluble Aβ_1-40_/Aβ_1-42_ (sAβ) levels^44^, we also measured sAβ levels following a single or repeated cycles of p300i treatment. Again, we found that both a single or two treatment courses (with an interval of 1 month in between) were effective in reducing cerebral sAβ, yet the effect observed 2 months following commencement of the treatment with p300i was stronger following repeated courses (Fig. 5h). These results indicated that while a single session of targeting Tregs is effective, repeated sessions of treatment would be advantageous for maintaining a long-lasting therapeutic effect, similar to our observations following weekly GA treatment (Supplementary Fig. 2h,i).

### Disease exacerbation by augmenting immune suppression

**Figure 6:**
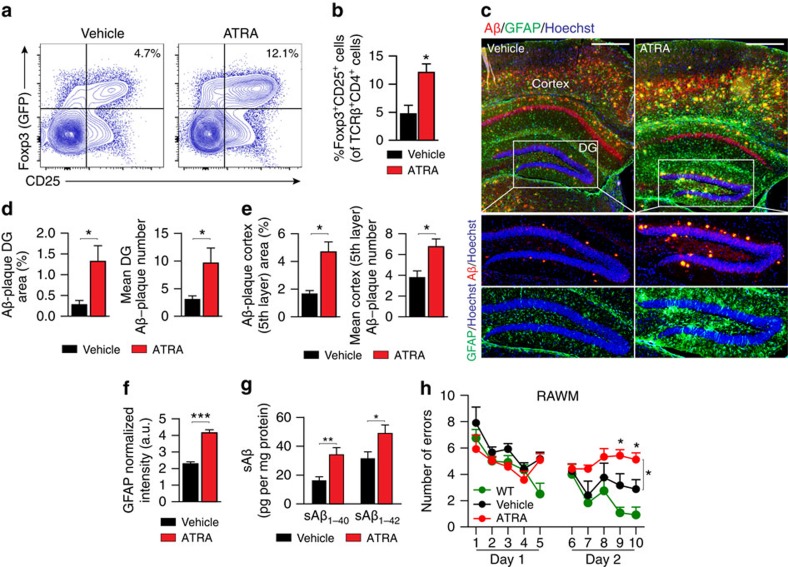
Augmenting systemic immune suppression in AD-Tg mice exacerbates disease pathology. (**a**,**b**) Representative flow cytometry plots (**a**), and quantitative analysis (**b**), showing elevation in frequencies of CD4^+^/Foxp3^+^/CD25^+^ Treg splenocytes in 5-month-old AD-Tg mice that received either ATRA or vehicle (DMSO) for a period of 1 week (*n*=5 per group; Student's *t*-test). (**c**–**f**) Representative microscopic images (**c**) and quantitative analysis (**d**–**f**) of Aβ plaque burden and astrogliosis in the brains of AD-Tg mice that were treated at the age of 5 months with either ATRA or vehicle (DMSO) for a period of 1 week, and subsequently examined after 3 additional weeks. Brains were immunostained for Aβ plaques (in red), GFAP (marking astrogliosis, in green) and by Hoechst nuclear staining (*n*=4–5 per group; Scale bar, 250 μm). Mean Aβ plaque area and plaque numbers were quantified in the hippocampal DG and the fifth layer of the cerebral cortex, and GFAP immunoreactivity was measured in the hippocampus (in 6 μm brain slices; *n*=5–6 per group; Student's *t*-test). (**g**) Levels of soluble Aβ_1–40_ and Aβ_1–42_, quantified by ELISA, in the cerebral brain parenchyma of AD-Tg mice, that were treated at the age of 5-months with either ATRA or vehicle (DMSO) for a period of 1 week, and subsequently examined after 3 additional weeks (*n*=5–6 per group; Student's *t*-test). (**h**) Cognitive performance in the RAWM task of AD-Tg mice, which were treated at the age of 5-months with either ATRA or vehicle (DMSO) for a period of 1 week, and subsequently examined after 3 additional weeks (*n*=5 per group; two-way repeated measures ANOVA followed by Bonferroni *post hoc* for individual pair comparisons). In all panels, error bars represent mean±s.e.m.; *P<0.05; **P<0.01; ***P<0.001.

Finally, to substantiate the negative role of Treg-mediated systemic immune suppression in AD, we further investigated the effect of increasing systemic Treg levels on AD pathology. Since adoptively transferred Tregs have the potential to downregulate their Foxp3 expression and become effector cells, especially when transferred *in vivo* into mice with abnormal adaptive immune system^45^, we adopted a pharmacological approach that enhances systemic immunosuppression. We treated AD-Tg mice by administration of all-trans retinoic acid (ATRA), which induces Treg differentiation^46^, stabilizes Treg phenotype^47^ and renders Tregs more suppressive^47^. We used 5-month-old 5XFAD AD-Tg mice, a relatively early stage of the disease in terms of cognitive impairments, to enable detection of worsening cognitive deficits, and treated these mice with either ATRA or vehicle (DMSO). A short administration protocol (every other day for 1 week) of ATRA was used, rather than continuous administration which can have a direct effect on the brain^48^. ATRA-treated AD-Tg mice showed significantly higher splenocyte frequencies of Foxp3^+^CD25^+^ Tregs (Fig. 6a,b). Examining the subsequent effect on disease pathology 3 weeks after the last ATRA injection revealed higher cerebral Aβ plaque burden and gliosis (approximately two- to threefold increase; Fig. 6c–f) and increased cerebral sAβ_1-40_ and sAβ_1-42_ levels (Fig. 6g). Assessment of cognitive performance of AD-Tg mice treated with ATRA, using the RAWM task, showed worsening of the spatial memory deficits, relative to vehicle-treated AD-Tg mice (Fig. 6h). Taken together, these results further substantiated the inverse correlation between systemic Treg-mediated immune suppression and AD progression.

## Discussion

Our study identifies systemic Foxp3^+^ Treg-mediated immunosuppression as a negative player in AD pathology, acting at least in part by reducing CP IFN-γ availability needed for gateway activity of this compartment in orchestrating recruitment of leukocytes to the CNS (refs 17, 18, 19, 25). Our findings also highlight the potential of breaking systemic immune tolerance as a novel target for AD immunotherapy.

Systemic Tregs are crucial for maintenance of autoimmune homeostasis and protection from autoimmune diseases^27^,^49^. Nevertheless, our findings suggest that under neurodegenerative conditions, when a reparative immune response is needed within the brain, the ability to mount this response is curtailed by systemic Tregs. This functional linkage between Tregs and AD pathology was demonstrated here by transient Treg depletion, using conditional genetic ablation of Foxp3^+^ cells. An inverse correlation between systemic immunosuppression and mitigation of AD pathology was also demonstrated using pharmacological approaches that interfere with Foxp3^+^ Treg cell activity or by systemic immunomodulation using GA or ATRA. In all of these approaches, we found that mitigation of pathology was associated with augmented systemic levels of IFN-γ-producing cells, and a tissue-specific effect on the CP in its ability to support leukocyte trafficking to the CNS. Notably, we found that during disease progression, in both 5XFAD and APP/PS1 AD-Tg mice, IFN-γ levels at the CP decrease. These findings are consistent with our previous reports of CP dysfunction in brain aging^25^,^26^, and in animal models of neurodegenerative diseases^19^,^20^, in which CP gateway activity for leukocyte trafficking diminishes. Under these conditions, CP activation for leukocyte trafficking was accompanied by a beneficial effect on CNS function, suggesting suboptimal trafficking of immune cells via the CP as an underlying mechanism shared in the pathophysiology of neurodegenerative conditions^22^. Since neuro-immunological cross-talk is an integral part of life-long brain plasticity^50^,^51^, and neurodegenerative diseases are predominantly age-related, these findings may also suggest a more general phenomenon, in which systemic immune suppression interferes with brain function.

Outside the CNS, it is generally accepted that immune suppression could curtail tissue-specific immune surveillance^52^. This concept is best exemplified in cancer immunotherapy, in which clinical efforts are made to unleash the power of the immune system to eradicate tumours^38^,^39^,^53^. Given the structure of the CNS as an immune privileged site, which is shielded behind barriers from direct interaction with the immune system, these ideas have not been favourably considered in brain pathologies. Previous research investigating the role of Tregs in CNS repair has yielded contradictory conclusions, with both protective and destructive functions documented^54^,^55^,^56^,^57^,^58^,^59^,^60^. Here we describe how systemic reduction in peripheral Tregs is subsequently followed by their accumulation in the CNS, which may suggest that peripheral and tissue-infiltrating Tregs play distinct roles in brain pathology. In line with recent findings studying Treg involvement in acute injuries of the CNS^59^,^60^, we found that timely and transient depletion of Tregs is associated with a neuroprotective effect in AD-Tg mice.

Our findings support the notion that under neurodegenerative conditions recruitment of immunoregulatory cells to CNS sites of pathology is essential for mitigating the neuroinflammatory response^22^,^23^. Here we found that this process involves accumulation of mo-MΦ and Tregs at cerebral sites of plaque formation, suggesting an active anti-inflammatory role for these immune cell populations. It is yet to be determined, however, whether the recruited immune cells play a direct role in Aβ phagocytosis or whether they induce phagocytic activity of other cell types, such as microglia^10^,^61^. Previous studies of immune cell-mediated Aβ phagocytosis have supported both of these possibilities. Moreover, the transient reduction in systemic Treg levels or activities may contribute to disease mitigation via additional mechanisms, including supporting a CNS-specific protective autoimmune response^23^,^62^ or augmenting the level of circulating monocytes that play a role in clearance of vascular Aβ^63^.

IL-10 is the major cytokine through which Tregs maintain their immunosuppressive activities^64^. Therefore the beneficial effect of the transient reduction of peripheral Tregs could be attributed to reduction in IL-10, and might shed new light on recent studies that demonstrated a detrimental role of this anti-inflammatory cytokine in AD pathology; APP/PS1 AD-Tg mice with IL-10 deficiency were found to display reduced cerebral Aβ plaque load and reduced synaptic and cognitive deficits^65^, while chronic overexpression of IL-10 in TgCRND8 AD-Tg mice, which induced IL-10 elevation in both the CNS and plasma, exacerbated disease pathology^66^. Notably, here we observed that within the brain, the resolution of the neuroinflammatory response was associated with local elevation of IL-10, alongside downregulation of pro-inflammatory cytokines, such as IL-12p40 and TNF-α. These results are consistent with our recent findings in a mouse model of Amyotrophic lateral sclerosis (ALS) disease, showing that recruitment of IL-10-expressing immunomodulatory cells to the CNS is associated with slower progression of ALS-like disease and prolonged life-span of the mice^20^. Together, these results highlight the need to distinguish between systemic and local contributions of IL-10 in brain pathologies.

Finally, though neurodegenerative diseases of different aetiology share a common local neuroinflammatory component, our results strongly argue against simplistic characterization of all CNS pathologies as diseases that would uniformly benefit from systemic anti-inflammatory therapy. Our findings further imply that periodic courses of treatment to reduce systemic immune suppression may represent a therapeutic or even preventive approach, applicable to a wide range of brain pathologies, including AD and age-associated dementia.

## Methods

### Animals

Heterozygous 5XFAD transgenic mice (Tg6799; on a C57/BL6-SJL background) that co-overexpress familial AD mutant forms of human APP (the Swedish mutation, K670N/M671L; the Florida mutation, I716V; and the London mutation, V717I) and PS1 (M146L/L286V) transgenes under transcriptional control of the neuron-specific mouse Thy-1 promoter^24^, and AD double transgenic B6.Cg-Tg (APPswe, PSEN1dE9) 85Dbo/J mice^67^ (on a C57BL/6 background) were purchased from The Jackson Laboratory. Genotyping was performed by PCR analysis of tail DNA, as previously described^24^. Heterozygous mutant *cx*_*3*_*cr1*^GFP/+^ mice^68^ (on a C57BL/6 background; B6.129P-*cx3cr1*^*tm1Litt*^/J, in which one of the CX_3_CR1 chemokine receptor alleles was replaced with a gene encoding GFP) were used as donors for BM chimeras. Foxp3.LuciDTR mice^69^ (on a C57BL/6 background) were bred with 5XFAD mice to enable conditional depletion of Foxp3^+^ Tregs. Male and female mice were bred and maintained by the Animal Breeding Center of the Weizmann Institute of Science. Age of mice is indicated for each experiment in figure legends. All experiments detailed herein complied with the regulations formulated by the Institutional Animal Care and Use Committee (IACUC) of the Weizmann Institute of Science.

### RNA purification and RT-qPCR analysis

Total RNA of the hippocampal DG was extracted with TRI Reagent (Molecular Research Center) and purified from the lysates using an RNeasy Kit (Qiagen). Total RNA of the CP was extracted using an RNA MicroPrep Kit (Zymo Research). mRNA (1 μg) was converted into complementary DNA (cDNA) using a High Capacity cDNA Reverse Transcription Kit (Applied Biosystems). The expression of specific mRNAs was assayed using fluorescence-based RT-qPCR. RT-qPCR reactions were performed using Fast-SYBR PCR Master Mix (Applied Biosystems). Quantification reactions were performed in triplicate for each sample using the standard curve method. Peptidylprolyl isomerase A (*ppia*) was chosen as a reference (housekeeping) gene. The amplification cycles were 95 °C for 5 s, 60 °C for 20 s and 72 °C for 15 s. At the end of the assay, a melting curve was constructed to evaluate the specificity of the reaction. For *ifn-γ* and *ppia* gene analysis, the cDNA was pre-amplified in 14 PCR cycles with non-random PCR primers, thereby increasing the sensitivity of the subsequent real-time PCR analysis, according to the manufacturer's protocol (PreAmp Master Mix Kit; Applied Biosystems). mRNA expression was determined using TaqMan RT-qPCR, according to the manufacturer's instructions (Applied Biosystems). All RT-qPCR reactions were performed and analysed using StepOne software V2.2.2 (Applied Biosystems). The TaqMan Assays-on-Demand probes Mm02342430_g1 (*ppia*) and Mm01168134_m1 (*ifn-γ*) were used.

For all other genes examined, the following primers were used:

*ppia*: forward 5′- AGCATACAGGTCCTGGCATCTTGT -3′ and reverse 5′- CAAAGACCACATGCTTGCCATCCA -3′;

*icam1*: forward 5′- AGATCACATTCACGGTGCTGGCTA -3′ and reverse 5′- AGCTTTGGGATGGTAGCTGGAAGA -3′;

*vcam1*: forward 5′- TGTGAAGGGATTAACGAGGCTGGA -3′ and reverse 5′- CCATGTTTCGGGCACATTTCCACA -3′;

*cxcl10*: forward 5′- AACTGCATCCATATCGATGAC -3′ and reverse 5′- GTGGCAATGATCTCAACAC -3′;

*ccl2*: forward 5′- CATCCACGTGTTGGCTCA -3′ and reverse 5′- GATCATCTTGCTGGTGAATGAGT -3′;

*tnf-α*: forward 5′- GCCTCTTCTCATTCCTGCTT -3′ reverse CTCCTCCACTTGGTGGTTTG -3′;

*il-1β*: forward 5′- CCAAAAGATGAAGGGCTGCTT -3′ and reverse 5′- TGCTGCTGCGAGATTTGAAG -3′;

*il-12*p40: forward 5′- GAAGTTCAACATCAAGAGCA -3′ and reverse 5′- CATAGTCCCTTTGGTCCAG -3′;

*il-10*: forward 5′- TGAATTCCCTGGGTGAGAAGCTGA -3′ and reverse 5′- TGGCCTTGTAGACACCTTGGTCTT -3′;

*tgfβ2*: forward 5′- AATTGCTGCCTTCGCCCTCTTTAC -3′ and reverse 5′- TGTACAGGCTGAGGACTTTGGTGT -3′;

*igf-1*: forward 5′- CCGGACCAGAGACCCTTTG and reverse 5′- CCTGTGGGCTTGTTGAAGTAAAA -3′;

*bdnf*: forward 5′- GATGCTCAGCAGTCAAGTGCCTTT -3′ and reverse 5′- GACATGTTTGCGGCATCCAGGTAA -3′;

### Immunohistochemistry

Tissue processing and immunohistochemistry were performed on paraffin-embedded sectioned mouse (6-μm thick) and human (10-μm thick) brains. For human ICAM-1 staining, primary mouse anti-ICAM (1:20 Abcam; ab2213) antibody was used. Slides were incubated for 10 min with 3% H_2_O_2_, and a secondary biotin-conjugated anti-mouse antibody was used, followed by biotin/avidin amplification with Vectastain ABC kit (Vector Laboratories). Subsequently, DAB (3,3′-diaminobenzidine substrate) (Zytomed kit) was applied; slides were dehydrated and mounted with xylene-based mounting solution. For tissue staining, mice were transcardially perfused with PBS prior to tissue excision and fixation. CP tissues were isolated under a dissecting microscope (Stemi DV4; Zeiss) from the lateral, third and fourth ventricles of the brain. For whole mount CP staining, tissues were fixed with 2.5% paraformaldehyde for 1 h at 4 °C, and subsequently transferred to PBS containing 0.05% sodium azide. Prior to staining, the dissected tissues were washed with PBS and blocked (20% horse serum, 0.3% Triton X-100 and PBS) for 1 h at room temperature. Whole mount staining with primary antibodies (in PBS containing 2% horse serum and 0.3% Triton X-100), or secondary antibodies, was performed for 1 h at room temperature. Each step was followed by three washes in PBS. The tissues were applied to slides, mounted with Immu-mount (9990402, from Thermo Scientific) and sealed with cover-slips. For staining of brain sections, two different tissue preparation protocols (paraffin-embedded or microtomed free-floating sections) were applied, as previously described^17^,^26^. The following primary antibodies were used: mouse anti-Aβ (1:300, Covance, #SIG-39320); rabbit anti-GFP (1:100, MBL, #598); rat anti-CD68 (1:300, eBioscience, #14-0681); rat anti-ICAM-1 (1:200, #AB2213); goat anti-GFP (1:100, Abcam, #ab6658); rabbit anti-IBA-1 (1:300, Wako, #019-19741); goat anti-IL-10 (1:20, R&D systems, Minneapolis, MN, #AF519); rat anti-Foxp3 (1:20, eBioscience, #13-5773-80); rabbit anti-CD3 (1:500, Dako, #IS503); mouse anti-ZO-1, mouse anti-E-Cahedrin and rabbit anti-Claudin-1 (all 1:100, Invitrogen, #33-9100, #33-4000, #51-9000); rabbit anti-GFAP (1:200, Dako, #Z0334). Secondary antibodies included: Cy2/Cy3/Cy5-conjugated donkey anti-mouse/goat/rabbit/rat antibodies (1:200; all from Jackson Immunoresearch). The slides were exposed to Hoechst nuclear staining (1:4,000; Invitrogen Probes) for 1 min. Two negative controls were routinely used in immunostaining procedures, staining with isotype control antibody followed by secondary antibody, or staining with secondary antibody alone. For Foxp3 intracellular staining, antigen retrieval from paraffin-embedded slides was performed using Retreivagen Kit (#550524, #550527; BD Pharmingen. Microscopic analysis was performed using a fluorescence microscope (E800; Nikon) or laser-scanning confocal microscope (LSM; Carl Zeiss, Inc.). The fluorescence microscope was equipped with a digital camera (DXM 1200F; Nikon), and with either a × 20 numerical aperture (NA) 0.50 or × 40 NA 0.75 objective lens (Plan Fluor; Nikon). The confocal microscope was equipped with LSM 510 laser-scanning capacity (three lasers: Ar 488, HeNe 543 and HeNe 633). Recordings were made on postfixed tissues using acquisition software (NIS-Elements, F3 (Nikon) or LSM). For quantification of staining intensity, total cell and background staining was measured using ImageJ software (NIH), and intensity of specific staining was calculated, as previously described^69^. Images were cropped, merged and optimized using Photoshop CS6 13.0 (Adobe), and were arranged using Illustrator CS5 15.1 (Adobe).

### Paraffin-embedded sections of human CP

Human brain sections of young and aged postmortem non-CNS-diseased individuals, as well as from AD patients, were obtained from the Oxford Brain Bank (formerly known as the Thomas Willis Oxford Brain Collection (TWOBC)) with appropriate consent and Ethics Committee approval (TW220). The experiments involving these sections were approved by the Weizmann Institute of Science Bioethics Committee.

### *In vitro* experiments

Following intracardial perfusion with PBS, the CPs were removed from the third, fourth, and lateral ventricles, under a dissecting microscope (Stemi DV4), and placed in tubes containing 0.25% trypsin (Life Technologies #15090-046). Tubes were shaken for 20 min at 37 °C, and the tissues were dissociated by manual pipetting. The cell suspension was washed in culture medium for epithelial cells (DMEM/HAM's F12 (Invitrogen Corp) supplemented with 10% FCS (Sigma-Aldrich), 1 mM l-glutamine, 1 mM sodium pyruvate, 100 U ml^−1^ penicillin, 100 mg ml^−1^ streptomycin, 5 μg ml^−1^ insulin, 20 μM Ara-C, 5 ng ml^−1^ sodium selenite and 10 ng ml^−1^ EGF), and cultured (2.5 × 10^5^ cells per well) at 37 °C, 5% CO_2_ in 24-well plates (Nunc). After 24 h, the medium was changed, and the cells were either left untreated or treated with the cytokines IFN-γ (10 ng ml^−1^) or IL-10 (10 ng ml^−1^) (Peprotech). For *ex vivo* differentiation of Tregs, splenocytes were collected from 5 to 8-week-old FoxP3-GFP mice, and following lysis of erythrocytes naive T cells were magnetically separated using a CD4^+^CD62L^+^ T-Cell Isolation Kit (Miltenyi Biotec, Germany). Cells were cultured (1 × 10^6^ cells per well) in complete RPMI 1640 medium w/o phenol red, supplemented with 10% FCS, 100 U ml^−1^ of penicillin, 100 mg ml^−1^ of streptomycin, 2 mM glutamine, 10 mM HEPES, 1 mM sodium pyruvate and 50 mM β-mercaptoethanol, all from biological industries (Beit Haemek, Israel). To drive differentiation of naive CD4^+^ T cells to a regulatory phenotype (Treg cells), growth medium was supplemented also with 5 ng ml^−1^ IL-2 and 10 ng ml^−1^ of recombinant human TGFb (both purchased from R&D systems). After 4 days in culture, FoxP3 expression was verified using flow cytometry of GFP-expressing cells, and the cells were added to the CP cultures in a 1:1 epithelial/Treg ratio. Following additional 24 h of co-culturing, T cells were discarded and RNA was isolated from the adherent CP epithelial cells using the ZR RNA MicroPrep kit according to the manufacturer's protocol.

### Flow cytometry sample preparation and analysis

Mice were transcardially perfused with PBS, and tissues were treated as previously described^25^,^26^. Brains were dissected, and the different brain regions were removed under a dissecting microscope (Stemi DV4) in PBS, and tissues were dissociated using the gentleMACS dissociator (Miltenyi Biotec). CP tissues were isolated from the lateral, third and fourth ventricles of the brain, incubated at 37 °C for 45 min in PBS (with Ca^2+^/Mg^2+^) containing 400 U ml^−1^ collagenase type IV (Worthington Biochemical Corporation), and then manually homogenized by pipetting. Spleens were mashed with the plunger of a syringe and treated with ACK (ammonium chloride potassium)-lysing buffer to remove erythrocytes. In all cases, samples were stained according to the manufacturers' protocols. All samples were filtered through a 70-μm nylon mesh, and blocked with anti-Fc CD16/32 (1:100; BD Biosciences). For intracellular staining of IFN-γ, the cells were incubated with para-methoxyamphetamine (10 ng ml^−1^; Sigma-Aldrich) and ionomycin (250 ng ml^−1^; Sigma-Aldrich) for 6 h, and Brefeldin-A (10 μg ml^−1^; Sigma-Aldrich) was added for the last 4 h. Intracellular labelling of cytokines was done with BD Cytofix/Cytoperm Plus fixation/permeabilization kit (cat. no. 555028). For Treg staining, an eBioscience FoxP3-staining buffer set (cat. no. 00-5523-00) was used. The following fluorochrome-labelled monoclonal antibodies were purchased from BD Pharmingen, BioLegend, R&D Systems or eBiosciences, and used according to the manufacturers' protocols: PE or Alexa Fluor 450-conjugated anti-CD4; PE-conjugated anti-CD25; PerCP-Cy5.5-conjugated or Brilliant-violet-conjugated anti-CD45; FITC-conjugated anti-TCRβ; APC-conjugated anti-IFN-γ; APC-conjugated anti-FoxP3. Cells were analysed on an LSRII cytometer (BD Biosciences) using FlowJo software. In each experiment, relevant negative control groups, positive controls and single-stained samples for each tissue were used to identify the populations of interest and to exclude other populations.

### Preparation of BM chimeras

BM chimeras were prepared as previously described^18^,^20^. In brief, gender-matched recipient mice were subjected to lethal whole-body irradiation (950 rad) while shielding the head. The mice were then injected i.v. with 5 × 10^6^ BM cells from CX_3_CR1^GFP/+^ donors. Mice were left for 8–10 weeks after BM transplantation to enable reconstitution of the haematopoietic lineage, prior to their use in experiments. The percentage of chimerism was determined by flow cytometry analysis of blood samples according to percentages of GFP-expressing cells out of circulating monocytes (CD11b^+^). In this head-shielded model, an average of 60% chimerism was achieved, and CNS-infiltrating GFP^+^ myeloid cells were verified to be CD45^high^/CD11b^high^, representing mo-MΦ and not microglia^18^.

### Morris water maze

Mice were given three trials per day, for 4 consecutive days, to learn to find a hidden platform located 1.5 cm below the water surface in a pool (1.1 m in diameter). The water temperature was kept between 21 and 22 °C. Water was made opaque with milk powder. Within the testing room, only distal visual shape and object cues were available to the mice to aid in location of the submerged platform. The escape latency, that is, the time required to find and climb onto the platform, was recorded for up to 60 s. Each mouse was allowed to remain on the platform for 15 s and was then removed from the maze to its home cage. If the mouse did not find the platform within 60 s, it was manually placed on the platform and returned to its home cage after 15 s. The inter-trial interval for each mouse was 10 min. On day 5, the platform was removed, and mice were given a single trial lasting 60 s without available escape. On days 7 and 8, the platform was placed in the quadrant opposite the original training quadrant, and the mouse was retrained for three sessions each day. Data were recorded using the EthoVision V7.1 automated tracking system (Noldus Information Technology). Statistical analysis was performed using analysis of variance (ANOVA) and the Bonferroni *post hoc* test. All cognitive tasks were performed between 10:00 and 17:00 hours during the lights-off phase.

### Radial-arm water maze

The RAWM task was used to test spatial learning and memory, as was previously described in detail^71^. Briefly, six stainless-steel inserts were placed in the tank, forming six swim arms radiating from an open central area. The escape platform was located at the end of one arm (the goal arm), 1.5 cm below the water surface and in a pool 1.1 m in diameter. The water temperature was kept between 21 and 22 °C. Water was made opaque with milk powder. Within the testing room, only distal visual shape and object cues were available to the mice to aid in location of the submerged platform. The goal arm location remained constant for a given mouse. On day 1, mice were trained for 15 trials (spaced over 3 h), with trials alternating between a visible and hidden platform, and the last 4 trails with hidden platform only. On day 2, mice were trained for 15 trials with the hidden platform. Entry into an incorrect arm, or failure to select an arm within 15 s, was scored as an error. Spatial learning and memory were measured by counting the number of arm entry errors or the escape latency of the mice on each trial. Training data were analysed as the mean errors or escape latency, for training blocks of three consecutive trials.

### GA administration

Each mouse was s.c. injected with a total dose of 100 μg of GA (batch no. P53640; Teva Pharmaceutical Industries, Petah Tiqva, Israel) dissolved in 200 μl of PBS. Mice were either injected according to a weekly GA regimen^33^ (Supplementary Fig. 2a) or daily-GA administration (Fig. 4a). Mice were killed either 1 week after the last GA injection, or 1 month after treatment, as indicated for each experiment.

### Conditional ablation of Treg

DTx (8 ng g^−1^ body weight; Sigma) was injected i.p. daily for 4 consecutive days to Foxp3.LuciDTR mice^69^. The efficiency of DTx was confirmed by flow cytometry analysis of immune cells in the blood and spleen, achieving almost complete (>99%) depletion of the GFP-expressing FoxP3^+^ CD4^+^ Treg cells (Supplementary Fig. 1a).

### P300 inhibitor administration

Inhibition of p300 in mice was performed similar to a previously described protocol^43^. p300i (C646; Tocris Bioscience) was dissolved in DMSO and injected i.p. daily (8.9 mg kg^−1^ per day, i.p.) for 1 week. Vehicle-treated mice were similarly injected with DMSO.

### ATRA treatment

ATRA administration to mice was performed similar to a previously described protocol^60^. ATRA (Sigma) was dissolved in DMSO and injected i.p. (8 mg kg^−1^ per day) every other day over the course of 1 week. Vehicle-treated mice were similarly injected with DMSO.

### Aβ plaque quantitation

From each brain, 6-μm coronal slices were collected, and eight sections per mouse, from four different pre-determined depths throughout the region-of-interest (DG or cerebral cortex), were immunostained. Histogram-based segmentation of positively stained pixels was performed using the Image-Pro Plus software (Media Cybernetics, Bethesda, MD, USA). The segmentation algorithm was manually applied to each image, in the DG area or in the cortical layer V, and the percentage of the area occupied by total Aβ immunostaining was determined. Plaque numbers were quantified from the same 6-μm coronal brain slices, and are presented as average number of plaques per brain region. Prior to quantification, slices were coded to mask the identity of the experimental groups, and plaque burden was quantified by an observer blinded to the origin of the sample.

### sAβ protein isolation and quantification

Tissue homogenization and sAβ protein extraction was performed as previously described^72^. Briefly, cerebral brain parenchyma was dissected, snap-frozen and kept at −80 °C until homogenization. Proteins were sequentially extracted from samples to obtain separate fractions containing proteins of differing solubility. Samples were homogenized in ice-cold tissue homogenization buffer, containing 250 mM of sucrose, 20 mM of Tris base, 1 mM of EDTA and 1 mM of ethylene glycol tetra-acetic acid (pH 7.4), using a ground glass pestle in a Dounce homogenizer. After six strokes, the homogenate was mixed 1:1 with 0.4% diethylamine (DEA) in a 100 mM NaCl solution before an additional six strokes, and then centrifuged at 135,000*g* at 4 °C for 45 min. The supernatant (DEA-soluble fraction containing extracellular and cytosolic proteins) was collected and neutralized with 10% of 0.5Mof Tris-HCl (pH 6.8). Aβ_1-40_ and Aβ_1-42_ were individually measured by ELISA from the soluble fraction using commercially available kits (Biolegend; #SIG-38954 and #SIG-38956, respectively) according to the manufacturer's instructions.

### Statistical analysis

The specific tests used to analyse each set of experiments are indicated in the figure legends. Data were analysed using a two-tailed Student's *t*-test to compare between two groups, one-way ANOVA was used to compare several groups, followed by the Newman–Keuls *post hoc* procedure for pairwise comparison of groups after the null hypothesis was rejected (*P*<0.05). Data from behavioural tests were analysed using two-way repeated-measures ANOVA, and Bonferroni *post hoc* procedure was used for follow-up pairwise comparison. Sample sizes were chosen with adequate statistical power based on the literature and past experience, and mice were allocated to experimental groups according to age, gender and genotype. Investigators were blinded to the identity of the groups during experiments and outcome assessment. All inclusion and exclusion criteria were pre-established according to the IACUC guidelines. Results are presented as mean±s.e.m. In the graphs, *y*-axis error bars represent s.e.m. Statistical calculations were performed using GraphPad Prism software (GraphPad Software, San Diego, CA).

## Additional information

**How to cite this article:** Baruch, K. *et al*. Breaking immune tolerance by targeting Foxp3^+^ regulatory T cells mitigates Alzheimer's disease pathology. *Nat. Commun.* 6:7967 doi: 10.1038/ncomms8967 (2015).

## Supplementary Material

Supplementary Information Supplementary Figures 1-3
