# Supplementary material for: Breaking immune tolerance by targeting Foxp3+ regulatory T cells mitigates Alzheimer's disease pathology
Source: Nat Commun. 2015 Aug 18;6:7967. doi: 10.1038/ncomms8967 (PMC4557123; doi:10.1038/ncomms8967)
Supplement: Supplementary Information — Supplementary Figures 1-3 [file ncomms8967-s1.pdf]

Supplementary Fig. 1

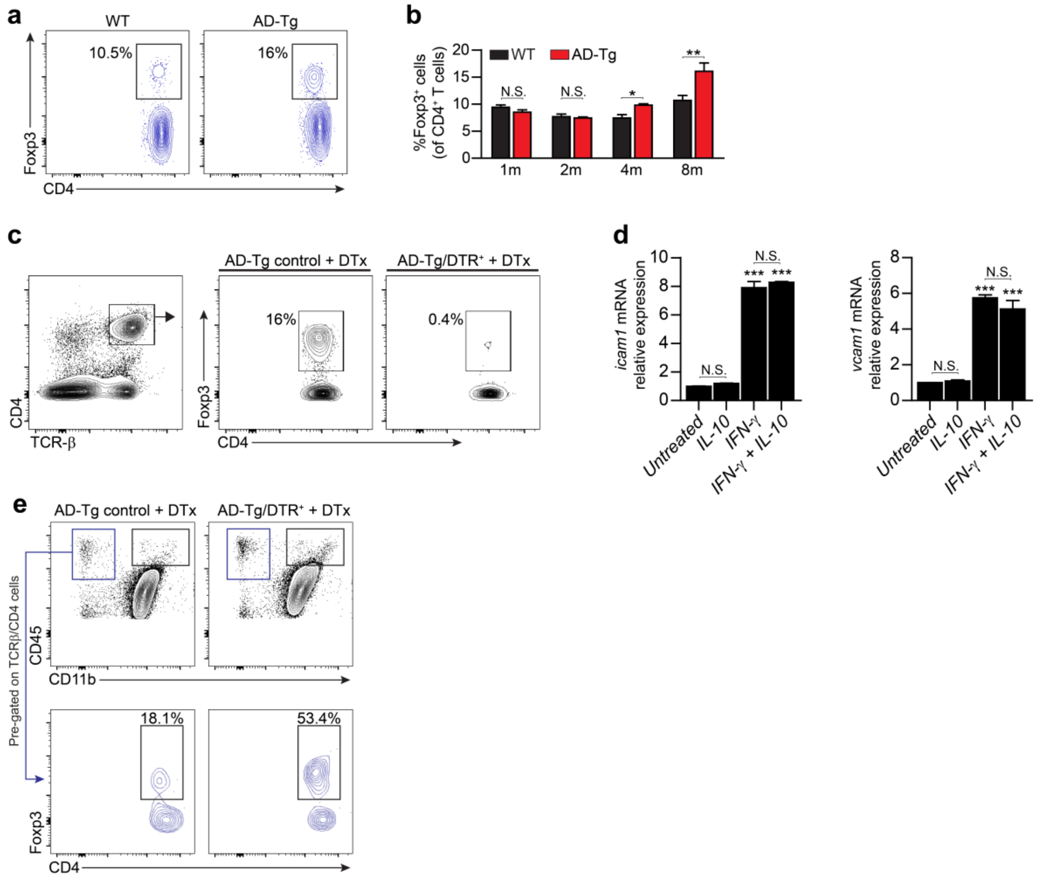

Supplementary Fig. 1. (**a, b**) Representative flow cytometry plots of CD4$^+$Foxp3$^+$ splenocyte frequencies (pre-gated on TCRβ) in 8-month old AD-Tg and WT control mice (a), and quantitative analysis of splenocytes from 1, 2, 4 and 8-month AD-Tg and WT control mice (b) (n=6-8 per group; Student's t test for each time point). (**c**) Gating strategy and representative flow cytometry plots of splenocytes from AD-Tg/Foxp3-DTR$^{+/-}$ mice, 1 day after the last injection of DTx. DTx was injected i.p. for 4 constitutive days, achieving ~99% depletion of Foxp3$^+$ cells. (**d**) mRNA levels of the genes *icam1* and *vcam1*, measured by RT-qPCR, in cultured CP cells from WT mice 24h after the addition of the cytokines IFN-γ (10 ng/ml), IL-10 (10 ng/ml), or their combination, relative to untreated (UT) cells (n=3 per group; ***, P < 0.001 versus untreated cells; one-way ANOVA followed by Newman–Keuls post hoc analysis; N.S., not significant). (**e**) Flow cytometry gating strategy for the data presented in **Fig. 2d-f**. In all panels, error bars represent mean ± s.e.m.; *, P < 0.05; **, P < 0.01;***, P < 0.001.

Supplementary Fig. 2

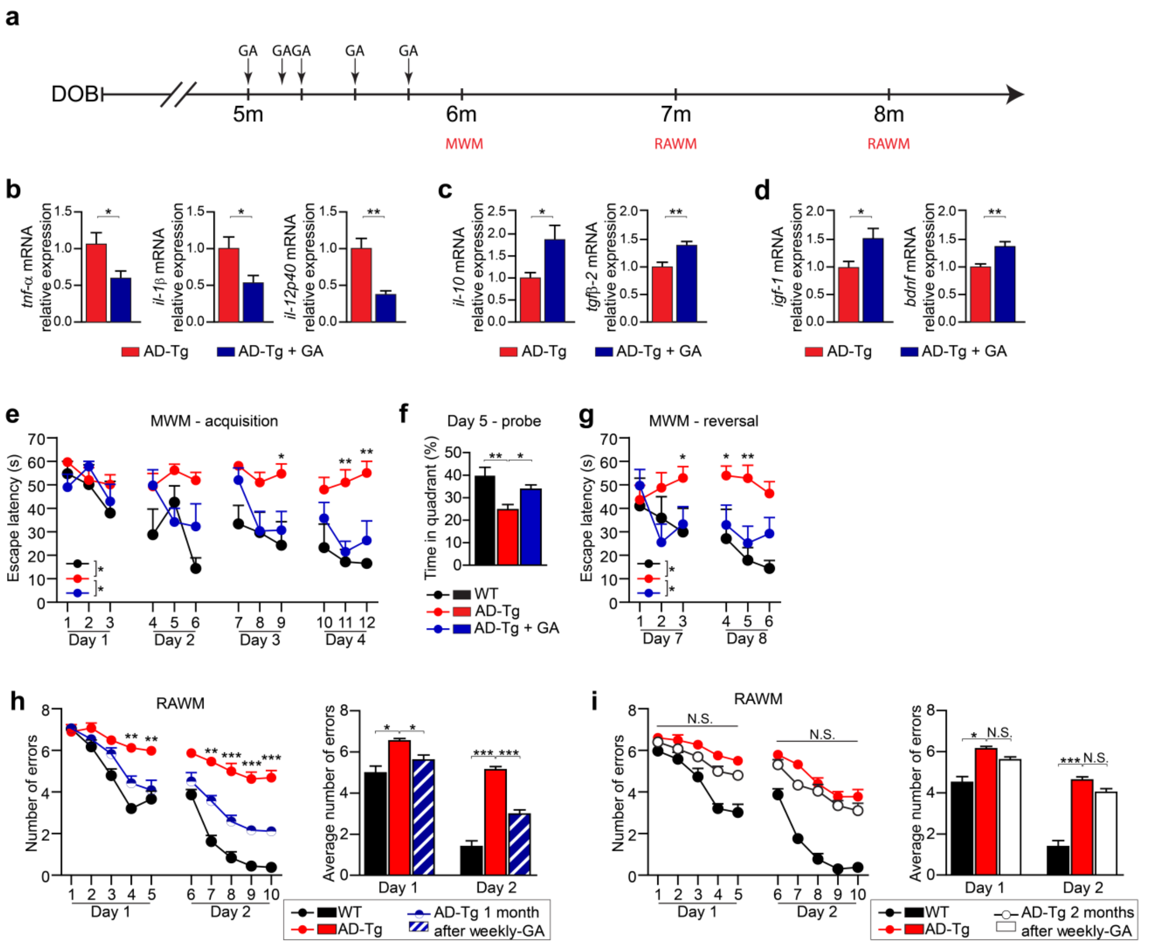

Supplementary Fig. 2. (**a**) Schematic representation of weekly-GA treatment regimen. Mice (5-months old) were s.c. injected with GA (100µg), twice during the first week (on day 1 and 4), and once every week thereafter, for an overall period of 4 weeks. The mice were examined for cognitive performance, 1 week (MWM), 1 month (RAWM) and 2 months (RAWM, using different experimental spatial settings) after the last injection, and for hippocampal inflammation. (**b-d**) mRNA expression levels of genes in the hippocampus of untreated AD-Tg mice (in red), and AD-Tg mice treated with weekly-GA (in blue), at the age of 6m, showing reduced expression of pro-inflammatory cytokines such as TNF-α, IL-1β and IL-12p40, and elevation of the anti-inflammatory cytokines IL-10 and TGF-β, and of the neurotropic factors, IGF-1 and BDNF, in weekly-GA treated mice (n=6-8 per group; Student's t test). **(e-g)** AD-Tg mice (5 months old) were treated with either weekly-GA or with vehicle (PBS), and compared to age-matched WT littermates in the MWM task at the age of 6m. Treated mice (blue line) showed

better spatial learning/memory performance in the acquisition (**e**), probe (**f**) and reversal (**g**) phases of the MWM, relative to controls (red line) (n=6-9 per group; two-way repeated measures ANOVA followed by Bonferroni post-hoc for individual pair comparisons). (**h-i**) Cognitive performance of the same mice in the RAWM task, 1 month (**h**) or 2 months (**i)** following the last GA injection (n=6-9 per group; two-way repeated measures ANOVA followed by Bonferroni post-hoc for individual pair comparisons). Data are representative of at least three independent experiments. In all panels, error bars represent mean ± s.e.m.; *, $P < 0.05$; **, $P < 0.01$;***, $P < 0.001$.

Supplementary Fig. 3

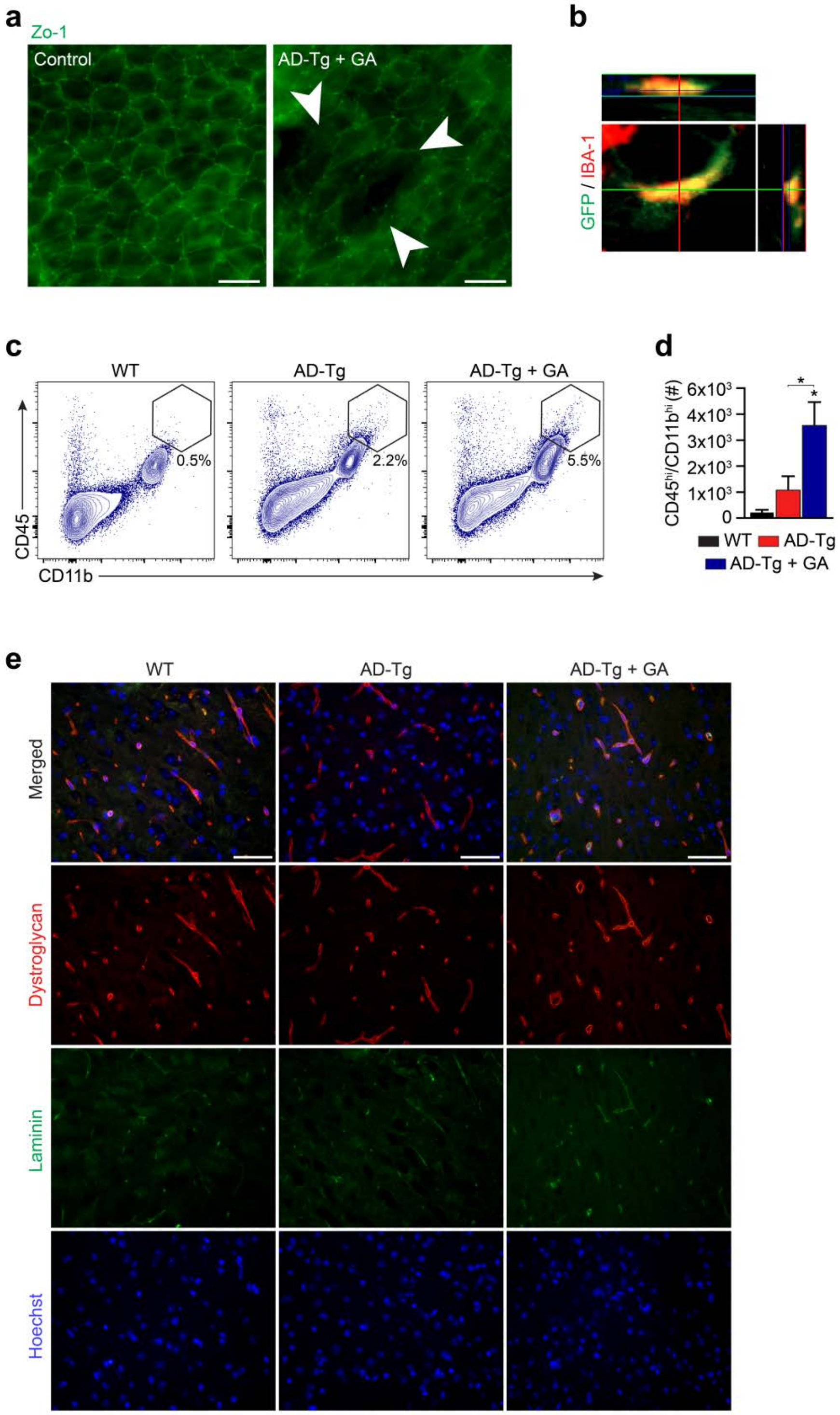

Supplementary Fig. 3. (**a**) Representative confocal images of CP from 7-month old AD-Tg mice, immunostained for ZO-1 to mark epithelial tight junctions, at the end of the 1st week of the weekly-GA regimen, as compared to untreated AD-Tg mice (scale bar, 50µm). Arrowheads indicate areas of disorganized tight junctions in the CP. (**b**) Orthogonal projection of confocal z-axis stacks, showing co-localization of GFP^+ cells (in green) with the myeloid marker, IBA-1 (in red) in the hippocampus of 7-month old weekly-GA treated AD-Tg mice. (**c, d**) Representative flow cytometry plots of cells isolated from the hippocampus of 4-month old WT, untreated AD-Tg, and AD-Tg mice, on the 2nd week of the weekly-GA regimen. $CD11b^{high}/CD45^{high}$ mo-MΦ were gated (**c**) and quantified (**d**) (n=4-5 per group; bars represent mean ± s.e.m.; *, $P < 0.05$; one-way ANOVA followed by Newman–Keuls post hoc analysis). (**e**) Representative microscopic images of the cerebral cortex of 8-month WT, untreated AD-Tg, and weekly-GA treated AD-Tg mice, immunostained for laminin (green) and dystroglycan (scale bar, 50µm).
